# A record of *Anzygina billi* Fletcher & Larivière, 2009 (Hemiptera: Cicadellidae) from New Zealand

**DOI:** 10.3897/BDJ.1.e954

**Published:** 2013-09-16

**Authors:** Stephen E. Thorpe

**Affiliations:** † School of Biological Sciences (Tamaki Campus), University of Auckland, Auckland, New Zealand

**Keywords:** *Anzygina billi*, Cicadellidae, Typhlocybinae, Australia, New Zealand, Auckland, NZOR, *Rubus*

## Abstract

The presence in New Zealand of the typhlocybine cicadellid *Anzygina billi* Fletcher & Larivière, 2009 is confirmed, based on new material from Auckland. *Rubus* sp. is confirmed as a host plant.

## Introduction

*Anzygina billi* Fletcher & Larivière, 2009 was described from Australia (Queensland), without indication of host plant. New Zealand was given as a possible additional locality for this species, based solely on fig. 3f in Knight (1976), considered by him to be a variant of *Anzygina dumbletoni* (Ghauri, 1963), a species which has *Rubus* sp. as a host plant. The uncertainty of New Zealand as a locality was because Knight did not specify any locality for the illustrated specimen (though it was in the context of a New Zealand revision), the relevant specimen(s) cannot now be located, and also because one important diagnostic character of *Anzygina billi* (aedeagus with a distinct posterior angulation) was not illustrated by Knight. Nevertheless, no other known species of *Anzygina* has an aedeagus like that depicted in fig. 3f of Knight (1976).

## Taxon treatments

### Anzygina billi

Fletcher & Larivière, 2009

#### Materials

- **Type status:** Other material. **Occurrence:** recordedBy: Stephen Thorpe; individualCount: 3; sex: males; **Location:** country: New Zealand; verbatimLocality: Coastal cliffs at Point England Reserve, Glen Innes, Auckland; verbatimElevation: 0-2 m; verbatimLatitude: 36.88048S; verbatimLongitude: 174.87598E; **Event:** eventDate: 1 January 2013; habitat: Rubussp. growing less than 2 metres above the beach; **Record Level:** institutionCode: Auckland Museum

#### Description

The only diagnostic morphological characters for *Anzygina billi* are afforded by the aedeagus, which comprises a single narrow shaft, tapering from base to apex, bearing two short apical processes. In lateral view, the shaft is distinctly angled posteriorly at approximately midlength. Fig. 3f in Knight (1976) depicts just such an aedeagus in posterior view. Knight did not illustrate it in lateral view, and fig. 3e is clearly a lateral view of the aedeagus depicted in fig. 3c, based on the relative length of the apical processes, though the wording in the figure caption somewhat misleadingly suggests that it corresponds instead to fig. 3f. Hence, it is unclear if the aedeagus depicted in fig. 3f was distinctly angled posteriorly in lateral view or not. Figs. 26 and 27 in Fletcher and Larivière (2009) depict the aedeagus of *Anzygina billi* in both views, although these figures seem somewhat schematic, and lack, for example, the detail seen in Knight's illustration of the shape of the apical processes. Nevertheless, Fletcher and Larivière (2009) claim that Knight's illustration matches the aedeagus of *Anzygina billi*, so, if material can be found which matches Knight's illustration, and which in addition has the shaft of the aedeagus distinctly angled posteriorly at approximately midlength, then this can be identified with a high level of confidence as *Anzygina billi*.

**Figure 1.**
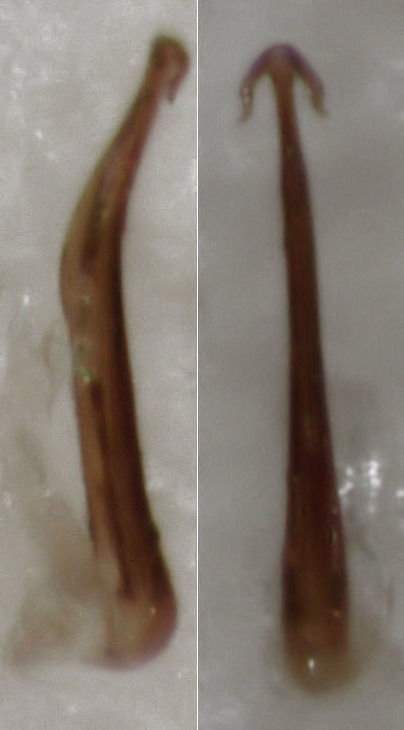
*Anzygina billi* aedeagus in lateral (left) and anterior (right) view (length about 0.26 mm).

On 1 January 2013, I examined some *Rubus* sp. (a large and complex genus, but here clearly not one of the native species) growing less than 2 metres above the beach on coastal cliffs at Point England Reserve, Glen Innes, Auckland. I observed several adults and exuviae of Typhlocybinae, and I collected three specimens for closer examination. All three specimens were males with identical aedeagus (see Fig. 1). One specimen will be vouchered intact in Auckland Museum (the aedeagus was visible without dissection while the specimen was fresh). The shape of the aedeagus in anterior/posterior aspect clearly matches fig. 3f in Knight (1976), and in lateral aspect is seen to be distinctly angled posteriorly at approximately midlength. I therefore identify the species as *Anzygina billi*, and it certainly keys out to *Anzygina billi* using the key provided by Fletcher and Larivière (2009). The fact that all three males examined by me had identical aedeagus strongly suggests that it is a constant feature and not some kind of individual aberration. Given that *Anzygina billi* is currently considered to be a good species, and no longer as a mere variant of *Anzygina dumbletoni*, I recommend that *Anzygina billi* be added to the New Zealand Organisms Register (NZOR) as present in the wild. Its "origin" is unclear, but I suggest that it be considered "exotic" with a question mark.

It is interesting to note that Fletcher and Larivière (2009) examined only two specimens of *Anzygina* from *Rubus* in Auckland, both identified by them as *Anzygina dumbletoni*. Both were claimed to be males, though the Mount Albert specimen was described as "missing except for tegmina and right hind tibia". It would be very difficult to recognise this specimen as a male, if this were the case, and impossible to identify it without the aedeagus, so I have my doubts about this record. Perhaps they meant to say that it was "missing tegmina and right hind tibia"? That would make more sense, but it is not what they said. It is not entirely clear if the aedeagus of the other specimen, from Henderson, was examined either. At any rate, with such a small amount of material of *Anzygina* from *Rubus* in Auckland, it is not surprising that *Anzygina billi* has been overlooked here, and, indeed, I suggest that further confirmation is needed that *Anzygina dumbletoni* occurs in Auckland, as evidence seems minimal at present. What is needed is an illustration of the aedeagus of material from Auckland.

## Supplementary Material

XML Treatment for Anzygina billi
